# Quality requirements for MRI simulation in cranial stereotactic radiotherapy: a guideline from the German Taskforce “Imaging in Stereotactic Radiotherapy”

**DOI:** 10.1007/s00066-023-02183-6

**Published:** 2024-01-02

**Authors:** Florian Putz, Michael Bock, Daniela Schmitt, Christoph Bert, Oliver Blanck, Maximilian I. Ruge, Elke Hattingen, Christian P. Karger, Rainer Fietkau, Johanna Grigo, Manuel A. Schmidt, Tobias Bäuerle, Andrea Wittig

**Affiliations:** 1 Strahlenklinik, Universitätsklinikum Erlangen, Friedrich-Alexander-Universität Erlangen-Nürnberg, Erlangen, Germany; 2 Klinik für Radiologie—Medizinphysik, Universitätsklinikum Freiburg, Freiburg, Germany; 3 Klinik für Strahlentherapie und Radioonkologie, Universitätsmedizin Göttingen, Göttingen, Germany; 4 Klinik für Strahlentherapie, Universitätsklinikum Schleswig-Holstein, Campus Kiel, Kiel, Germany; 5 Klinik für Stereotaxie und funktionelle Neurochirurgie, Zentrum für Neurochirurgie, Universitätsklinikum Köln, Cologne, Germany; 6 Institut für Neuroradiologie, Universitätsklinikum Frankfurt, Frankfurt am Main, Germany; 7 Abteilung Medizinische Physik in der Strahlentherapie, Deutsches Krebsforschungszentrum (DKFZ), Heidelberg, Germany; 8 Nationales Zentrum für Strahlenforschung in der Onkologie (NCRO), Heidelberger Institut für Radioonkologie (HIRO), Heidelberg, Germany; 9 Neuroradiologisches Institut, Universitätsklinikum Erlangen, Friedrich-Alexander-Universität Erlangen-Nürnberg, Erlangen, Germany; 10 Radiologisches Institut, Universitätsklinikum Erlangen, Friedrich-Alexander-Universität Erlangen-Nürnberg, Erlangen, Germany; 11 Klinik und Poliklinik für Strahlentherapie und Radioonkologie, Universitätsklinikum Würzburg, Würzburg, Germany

**Keywords:** MRI simulation, Stereotactic radiotherapy, Stereotactic radiosurgery, Brain metastases, Vestibular schwannoma, Meningioma, Trigeminal neuralgia, Pituitary adenoma, DEGRO, DGMP, DGNC, DGNR, DS-ISMRM, Guideline

## Abstract

Accurate Magnetic Resonance Imaging (MRI) simulation is fundamental for high-precision stereotactic radiosurgery and fractionated stereotactic radiotherapy, collectively referred to as stereotactic radiotherapy (SRT), to deliver doses of high biological effectiveness to well-defined cranial targets. Multiple MRI hardware related factors as well as scanner configuration and sequence protocol parameters can affect the imaging accuracy and need to be optimized for the special purpose of radiotherapy treatment planning. MRI simulation for SRT is possible for different organizational environments including patient referral for imaging as well as dedicated MRI simulation in the radiotherapy department but require radiotherapy-optimized MRI protocols and defined quality standards to ensure geometrically accurate images that form an impeccable foundation for treatment planning. For this guideline, an interdisciplinary panel including experts from the working group for radiosurgery and stereotactic radiotherapy of the German Society for Radiation Oncology (DEGRO), the working group for physics and technology in stereotactic radiotherapy of the German Society for Medical Physics (DGMP), the German Society of Neurosurgery (DGNC), the German Society of Neuroradiology (DGNR) and the German Chapter of the International Society for Magnetic Resonance in Medicine (DS-ISMRM) have defined minimum MRI quality requirements as well as advanced MRI simulation options for cranial SRT.

## Introduction

Magnetic Resonance Imaging (MRI) has a long-standing role in radiotherapy treatment planning for brain tumors and is essential today in nearly all cranial treatment indications [1]. Stereotactic Radiosurgery (SRS) and Fractionated Stereotactic Radiotherapy (FSRT), collectively referred to as Stereotactic Radiotherapy (SRT), demand exceptional precision to achieve doses of high biological effectiveness in tumors while simultaneously preserving the adjacent normal tissues and organs-at-risk (OARs) [2, 3]. This paradigm of cranial SRT has been shown to achieve high local cure rates with limited to minimal toxicities [4–6]. The stringent precision requirements of cranial SRT necessitates not only highly precise treatment planning and delivery, but also critically depends on optimal MRI for target volume definition. The previous German guideline on technological quality requirements for stereotactic radiotherapy explicitly pointed out that imaging for SRT treatment planning needs exceptional attention where “the target volume and all organs-at-risk are defined using organ-specific imaging modalities” and “secondary imaging requires accurate registration with the thin-slice planning computed tomography (CT)” [3]. This is especially important for MRI as an integral part of cranial SRT which most often forms the fundamental basis for all further treatment steps. Errors and uncertainties in MRI can often not be compensated at later stages in the treatment planning process and are propagated throughout the treatment planning chain, possibly leading to suboptimal treatment with inferior outcome. Multiple groups clearly demonstrated that inadequate MRI would affect the accuracy of gross target volume (GTV) delineation, which can easily diminish the clinical outcome [7–9]. Simply expanding the margins to compensate for uncertainties and imaging errors will increase the planning target volume (PTV), consecutively decrease the therapeutic selectivity, and will ultimately diminish the therapeutic principles of precision radiotherapy. Cranial stereotactic radiotherapy was outside the scope of recently published guidelines on MRI simulation in radiotherapy, like the AAPM task group 284 report [10, 11] and a comprehensive guideline on MRI simulation for cranial SRT was missing. Optimal MRI simulation and target volume definition frequently require interdisciplinary input and close collaboration, integrating radiooncologic, diagnostic, neurosurgical and physics expertise. In this guideline, therefore, an interdisciplinary panel including experts from the working group for radiosurgery and stereotactic radiotherapy of the German Society for Radiation Oncology (DEGRO), the working group for physics and technology in stereotactic radiotherapy of the German Society for Medical Physics (DGMP), the German Society of Neurosurgery (DGNC), the German Society of Neuroradiology (DGNR) and the German Chapter of the International Society for Magnetic Resonance in Medicine (DS-ISMRM) have defined minimum quality requirements as well as advanced simulation options for MRI in cranial SRT to increase its quality in clinical practice and ultimately improve treatment outcomes.

## Methods

To formulate this guideline paper, a cross-disciplinary expert task force was constituted. The task force contained experts from radiation oncology, medical physics, neurosurgery, neuroradiology, radiology and MRI physics. The task force conducted biweekly virtual meetings from December 2022 to July 2023. First a systematic literature review on the areas of geometric accuracy, sequence selection and optimization of sequence protocol parameters, contrast agent-related parameters, time interval between MRI simulation and treatment delivery as well as image registration and imaging in SRT position was performed. This literature review was revisited, evaluated, and refined through iterative discussions in the regular meetings. Subsequently, requirements and recommendations were derived from the literature review with consensus being optimized in a two-stage process. Following an initial round of voting, the proposed requirements and recommendations were re-evaluated and fine-tuned in interdisciplinary discussions before proceeding to a final voting round. Each requirement and recommendation was subjected to a vote on agreement (with possible responses: “yes,” “no,” or “abstention”) and, in the case of agreement, the category (“minimum requirement”, “additional recommendation”, or “optional”). Guideline statements that were classified as “minimum requirement” were considered mandatory for MRI simulation in SRT. “Additional recommendations” were defined as recommendations that should be applied for optimal MRI simulation but are not considered mandatory. Finally, “optional” statements provide advanced options that can be implemented by experienced centers. Consensus was quantified as the percentage of agreement, excluding abstentions. For each requirement and recommendation, the rates of consensus, abstention, and the votes on the statement category are provided.

## Requirements and recommendations

## 1. Geometric accuracy

### 1.1. General geometric accuracy

Distortions in MRI may result from multiple mechanisms that may compromise the precise delivery of treatment [12–15] and will be discussed in detail below. Generally, MRI distortions are nonlinear and unevenly distributed across image datasets [13, 16, 17]. Most hardware-related distortions occur in the periphery and least distortions near the isocenter of the magnet [18–21]. Therefore, with cranial SRT, hardware-related distortions are expected to occur near the cortical surface of the brain, when the head is placed at the isocenter of the magnet [13, 16]. Another source of distortions is patient-specific: for example, prominent distortions can appear near air-bone interfaces at the frontopolar and orbitofrontal cortex, in addition to the cranial aspects of the prefrontal cortex and the lateral and inferior portions of the temporal lobes [17, 22].

#### General geometric accuracy—Minimum requirements

- The position of the patient inside the MR scanner must be optimized, so that the center of the imaged volume is as close as possible to the magnet and gradient isocenter. *(Consensus: 100%, abstention: 0%; Minimum requirement: 100%)*
- An end-to-end test including MRI simulation must be performed yearly after commissioning and after changes to the SRT treatment planning chain in accordance with DIN 6864‑1. *(Consensus: 100%, abstention: 0%; Minimum requirement: 92%, additional recommendation: 8%)*
- The radiologic report must state that MRI sequences have been acquired for the purpose of SRT treatment planning and optimized for geometric accuracy. *(Consensus: 100%, abstention: 8%; Minimum requirement: 92%, additional recommendation: 8%)*

### 1.2. MRI distortion correction using prior knowledge

Two types of MRI distortions are most relevant to cranial stereotactic radiotherapy: distortions that are caused by gradient coil nonlinearities and distortions that originate from inhomogeneities of the main magnetic field (*B*_0_) [23]. The *B*_0_ inhomogeneities in turn are caused by residual imperfections in the main magnet and by the magnetic susceptibility differences in the tissues of the patient which lead to static field inhomogeneities [15, 23, 24]. These distortions can be substantially reduced using prior knowledge about the image acquisition process. Optimized parameter settings are selected for the MR image acquisition to produce the most geometrically accurate images possible. Remaining distortions in the images are corrected in a distortion correction post-processing step. MRI distortion correction using prior knowledge is the preferred approach to achieve geometric accuracy and will be discussed in detail in subsequent Sects. 1.2.1. and 1.2.2.

#### 1.2.1. Gradient nonlinearity-related distortion (Fig. 1a, b)

Gradient nonlinearity-related distortions are usually the most significant type of distortion in cranial MRI [12]. Spatial encoding in MRI is based on spatially linearly varying magnetic fields (so-called gradients) that are created by 3 independent gradient coils in *x, y*, and *z* direction [12, 13, 15, 23, 25, 26]. However, due to inevitable physical constraints (e.g., Maxwell’s equation) in gradient coil design, gradient fields deviate from their linear behavior with increasing distance to the center of the gradient coils (which is typically coincident with the magnet isocenter) [26, 27]. During image reconstruction, these gradient nonlinearities result in spatial distortions that increase with the distance from the isocenter [15, 18, 21, 27, 28]. Gradient nonlinearities are specific to every gradient coil and are therefore constant for a given MRI system [12, 13, 15, 18, 24, 27]. As gradient nonlinearities are linked to the absolute position of the gradient coil, image distortions will vary when the patient is positioned differently relative to the gradient fields [18, 24]. The amount of distortion from gradient nonlinearities therefore depends on the MRI system model (more specifically: the type of installed gradient coil) and the patient position relative to the isocenter. Image distortions can reach up to several millimeters at the brain periphery [18, 27].

**Fig. 1:**
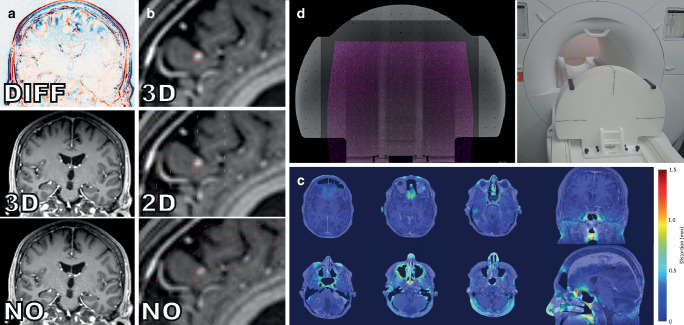
Importance of geometric accuracy for SRT treatment planning. **a** Influence of gradient non-linearity distortion correction. *Bottom*: uncorrected dataset, *center*: 3D-corrected dataset, *top*: difference map. **b** Influence of gradient non-linearity distortion correction in an exemplary patient with brain metastasis. *Arrows* indicate direction and magnitude of distortion. *Red outline*: Tumor perimeter in the 3D-corrected dataset. **c** Distortions due to magnetic susceptibility effects in an exemplary patient case acquired at 1.5 T. **d** Validation of geometric accuracy with MRI phantoms. *Left*: Ground truth phantom geometry obtained from CT (*grey scale*) overlayed by the MRI phantom acquisition (*magenta*), *Right*: External view of the phantom

Gradient nonlinearities are a fixed property of a given gradient coil type which are known by the manufacturer [26, 27]; thus, they can be corrected using a vendor-specific distortion correction. Vendor-specific gradient non-linearity distortion correction is usually implemented as a post-processing step using deformable registration (image warping), resampling and intensity correction [18, 27, 29]. Interestingly, gradient nonlinearity correction can be performed without having access to the MRI system: some MRI manufacturers offer distortion correction software that contains a library of distortion fields of all their gradient coils. By identifying the specific gradient coil in the DICOM metadata, distortions can be retrospectively corrected in any study acquired on one of their MRI systems. While vendors also offer a 2D distortion correction option, only 3D correction rectifies gradient nonlinearity-related distortion in all dimensions and therefore is the minimum requirement for SRT treatment planning.

The clinical impact of gradient nonlinearity-related distortions was investigated by Seibert et al. in cranial radiosurgery by comparing 3D corrected and uncorrected images. When uncorrected images were used, they found an average GTV displacement of 1.2 mm and a maximum GTV displacement of 3.9 mm. This would have resulted in 8 of 28 lesions experiencing geographic miss [9].

Residual distortion may remain after vendor-specific 3D correction. The amount of residual distortion should be characterized regularly with phantom measurements and corrected if necessary. Evaluating MRI geometric accuracy with phantom measurements is recommended during MRI system installation to obtain baseline data, and after major repair or and inspection work, which is typically scheduled at 6‑month intervals ([10, 30–32]; Fig. 1d). Multiple groups have reported how residual gradient nonlinearity distortions can be corrected by obtaining 3D deformation vector fields from phantom measurements [12, 26, 33]. Typically, a grid structure with known geometry is imaged and the deviation of the grid locations in the images from the known positions are quantified and extrapolated to the full image [34].

##### Gradient nonlinearity-related distortion—Minimum requirements

- Vendor 3D gradient nonlinearity distortion correction must be applied, when acquiring image datasets for SRT treatment planning. *(Consensus: 100%, abstention: 0%; Minimum requirement: 100%)*
- The residual gradient non-linearity-related distortions after vendor 3D correction should be characterized using phantom measurements for quality assurance at the time of commissioning, after scanner upgrades, repairs or maintenance, and at least in yearly intervals. The maximum amount of distortion obtained via phantom measurements for the field-of-view of a typical head MRI scan (sphere of 25 cm diameter) must not exceed 1 mm [30]. If larger distortion is present, this has to be addressed by arranging the repair of hardware or software components or by performing additional correction for the remaining image distortion. *(Consensus: 100%, abstention: 8%; Minimum requirement: 92%, additional recommendation: 8%)*

##### Gradient nonlinearity-related distortion—Additional recommendations

- Residual gradient non-linearity-related distortions after vendor correction should be characterized using phantom measurements for quality assurance in at least monthly intervals. This recommendation is derived from the ESTRO-ACROP guideline for online MRI guided radiotherapy systems and the ACR 2015 MRI quality control manual, which recommend monthly and weekly assessment of gradient non-linearity-related distortion, respectively [11, 32]. *(Consensus: 91%, abstention: 15%; Additional recommendation: 100%)*

##### Gradient nonlinearity-related distortion—Optional

- Consider, correcting residual distortions < 1 mm after vendor distortion correction based on phantom measurements to further minimize remaining distortion. *(Consensus: 92%, abstention: 0%; Optional: 100%)*

#### 1.2.2. Distortions due to *B*_0_ inhomogeneity and chemical shift

As with non-linearities of gradient coils, inhomogeneities of the main magnetic field (*B*_0_ inhomogeneities) also result in distortions. MRI *B*_0_ inhomogeneities are caused by imperfections in magnet design, but, more importantly, by magnetic perturbations induced by the patient [13]. Locally, *B*_0_ inhomogeneities cause static field gradients that are superimposed onto the dynamic image encoding gradients—thus, they affect both the slice selection and the readout process [13]. In standard 3D sequences, gradient nonlinearity-related displacements occur in all three dimensions, while *B*_0_ inhomogeneity-related distortions occur only in frequency-encoding (readout) direction. *B*_0_ inhomogeneities also affect the slice selection in 2D sequences resulting in non-ideal slice geometries that diverge from a perfectly planar configuration. Thus, 2D sequences are more susceptible to *B*_0_ inhomogeneity-induced distortions than 3D sequences [12, 13, 17].

Changes in magnetic susceptibility at tissue-tissue or air-tissue interfaces lead to patient-induced perturbations of the main magnetic field ([13, 23, 35]; Fig. 1c). Susceptibility-induced magnetic field distortions are directly proportional to the field strength—thus, larger distortions are expected at higher fields if the same imaging protocols are applied. The greatest susceptibility differences and, therefore, the largest distortions occur at air-bone interfaces and near metal implants [23, 35]. In cranial SRT, the most severe susceptibility-related distortions are expected near the paranasal sinuses and mastoid cells [17]. An analysis of a brain imaging protocol using a 3D gradient echo sequence with T1-weighted preparation (T1-MPRAGE) conducted in 2013 by Wang et al. revealed that susceptibility-induced distortions were < 0.5 mm in 86.9% of the imaged volume (3 T; using a rather low readout bandwidth of 180 Hz/pixel; patient-specific automated shimming activated). Although displacement averages were low for the entire imaged volume, sinus air-bone boundaries showed 1.6 mm of average distortion. Despite distortions degraded with distance from the sinuses, they extended into the adjacent brain and optic system, and still measured 0.8 mm at a distance of 12 mm, which is clinically relevant for stereotactic targets located close to the sinuses and mastoid cells [17]. Furthermore, large differences in susceptibility and, consequently, significant distortions can also occur near metallic implants, such as surgical clips [17]. It is possible to reduce distortions due to *B*_0_ inhomogeneities by increasing the readout bandwidth, by using 3D rather than 2D sequences [36], and by activating patient-specific active shimming.

Shim coils create additional magnetic fields that can partially compensate both system-related and patient-induced *B*_0_ inhomogeneities. Shim coils are typically installed in the MR magnet bore, or, additionally, in specialized RF coils to provide a more local field optimization. During shimming, at first the *B*_0_ inhomogeneities are measured using a fast field mapping protocol, followed by the calculation of the shim coil currents needed to minimize the field distortion in a given target region [17, 23, 37, 38]. Shimming is typically an iterative procedure which converges rapidly. In active shimming on 2D multi-slice MRI the shim currents are adapted to each slice separately—however, not all MRI systems support this feature. Even though shimming can improve the geometric accuracy during imaging, it should be used with care, as the local distortion fields will change after each re-adjustment of the shim currents. This can be especially challenging for radiotherapy applications, in which imaging protocols are used that automatically perform a shim (e.g., in diffusion-weighted echo planar (EPI) imaging).

Patient-specific active shimming and RT-optimized bandwidth settings (see below) may not be entirely effective in minimizing *B*_0_ inhomogeneity-related distortions. If additional correction is required, advanced techniques have been described that involve acquiring an improved higher resolution *B*_0_ map with the patient in the scanner that subsequently can be used for further rectification of patient-induced distortions via image post-processing [15, 39]. Reverse gradient methods have also been proposed to correct *B*_0_ inhomogeneity-related distortions but require obtaining every sequence twice with the opposite frequency encoding direction [30].

Furthermore, because wear and tear of individual components, incorrect software settings and small metallic objects such as ear pins left inside the magnet can cause unnoticed distortions [30, 36], regular quality assurance is recommended to ensure optimal images for stereotactic radiotherapy ([30]; Table 3). It is recommended to evaluate the main magnetic field homogeneity after installation (baseline), after each repair and maintenance session at the scanner, and at regular intervals, with a minimum frequency of at least once per year. To prevent the introduction of small metallic objects and dust into the scanner bore that could degrade magnetic field homogeneity and cause subtle distortion [36] it is essential to establish appropriate standard operating procedures. In addition, some MR scanner manufacturers offer a daily quick check of main magnetic field homogeneity for daily quality assurance. Moreover, gradient echo-based localizer images also offer an efficient means of screening for metal-induced distortions in each patient before acquiring sequences for radiotherapy treatment simulation. This MRI simulation quality assurance process is well complemented by an end-to-end test that encompasses MR imaging ([40], Table 3). Chemical shifts, such as the fat-water shift, also belong to the group of patient-related distortions. In the case of the fat-water shift, the different resonance frequencies of fat and water cause a shift of the fat-containing tissue along the frequency encoding direction [41].

##### Role of the main magnetic field strength (*B*_0_)

In general, there is an ongoing debate which magnetic field strength is best suited for RT applications [24]. With increasing *B*_0_ also the SNR in the images increases so that smaller lesions can be better detected. On the other hand, the tissue parameters T1 and T2 change with field strength and the imaging protocols need to be adapted to achieve the same contrast [42, 43]. Often similar gradient systems are used at 1.5 T and 3 T so that gradient-related distortions are comparable. Chemical shifts and susceptibility-related distortions on the other hand are proportional to *B*_0_ demanding higher readout bandwidths (i.e., stronger readout gradients) to minimize their effect. Thus, the SNR benefit of higher fields is often partially compensated by the need to acquire geometrically accurate images: with increasing readout bandwidth, all distortions resulting from *B*_0_ inhomogeneities decrease reciprocally [17, 23, 37, 38], but the SNR is inversely correlated with the square root of the read-out bandwidth [38]. In order to reduce distortions caused by *B*_0_ inhomogeneities, radiotherapy planning usually requires a field strength dependent higher read-out bandwidth than routine diagnostic imaging [24]. These higher readout bandwidths will also minimize chemical shifts (see above) [41].

The loss of SNR caused by a higher read-out bandwidth can be compensated by increasing the measurement time, optimizing the coil selection, and reducing motion artifacts with immobilization. Despite that 3 T scanners would be expected to suffer more from distortions related to *B*_0_ inhomogeneity, the profound SNR increase associated with higher field strengths also enables larger compensatory readout bandwidths. MR scanners operating at 3 T, therefore, generally can also be suitable for acquiring simulation MRI studies for stereotactic radiotherapy treatment planning [12, 37, 44].

More recently, MRI systems with 0.35 T and less have been built which are combined with a RT system for online tumor imaging. The suitability of these low-field systems for real-time tumor tracking has been demonstrated, but their use in treatment of small brain targets still needs further evaluation [45, 46].

##### Distortions due to B0 inhomogeneity and chemical shift—Minimum requirements

- The pixel bandwidth must be set to at least 440 Hz (i.e., twice the fat-water shift at 1.5 T) [10]. *(Consensus: 92%, abstention: 0%; Minimum requirement: 100%)*
- Active shimming must be used to actively mitigate magnetic field inhomogeneities from system imperfections and susceptibility-related inhomogeneities caused by the patient anatomy. *(Consensus: 100%, abstention: 0%; Minimum requirement: 100%)*
- The main magnetic field homogeneity must be characterized after installation (baseline), after scanner upgrades, repairs or maintenance, and at least in yearly intervals, as detailed in sources such as [32] and [33]. If necessary, arrange for repairs to maintain field homogeneity and ensure necessary geometric accuracy. *(Consensus: 100%, abstention: 0%; Minimum requirement: 100%)*
- Standard operating procedures must be established to minimize the introduction of small metallic objects (e.g., hairpins) and metallic dust (e.g., from shoes) into the scanner bore that could degrade magnetic field homogeneity and geometric accuracy. *(Consensus: 100%, abstention: 0%; Minimum requirement: 100%)*
- Screening for metallic objects inside the scanner bore that could degrade magnetic field homogeneity and geometric accuracy must be performed daily. In every patient, check for metal artifacts in gradient echo-based localizer images before acquiring images for treatment planning. *(Consensus: 100%, abstention: 0%; Minimum requirement: 100%)*

##### Distortions due to B0 inhomogeneity and chemical shift—Optional

- Consider, increasing the pixel bandwidth to at least 660 Hz to further reduce distortions due B0 inhomogeneities and chemical shift (i.e., three-times the fat-water shift at 1.5 T). Increasing the pixel bandwidth might entail more averages need to be acquired to preserve SNR and lesion conspicuity. *(Consensus: 92%, abstention: 8%; Optional: 100%)*
- Consider, individually characterizing main magnetic field inhomogeneities from system imperfections and susceptibility-related inhomogeneities for patients undergoing MRI simulation by using B0 mapping. *(Consensus: 92%, abstention: 8%; Optional: 100%)*
- Consider, individually correcting residual distortions because of magnetic field inhomogeneities based on individual B0 mapping or reverse gradient methods. *(Consensus: 100%, abstention: 8%; Optional: 100%)*

### 1.3. Distortion correction using image registration

Registration-based distortion correction is a generic method to reduce MR image distortion, but inferior to the prior knowledge-based methods discussed above. With registration-based distortion correction, MRI distortions are reduced via a specialized non-rigid registration to a planning CT. This correction method does not require any prior knowledge about the MRI acquisition process, but it estimates a 3D deformation field via registration to the planning CT which is assumed to be geometrically correct. Registration-based distortion correction was able to improve distortions in past analyses, albeit some studies only analyzed phantom data but no clinical cases [47, 48].

Registration-based distortion correction has some principal limitations: 1) the accuracy of non-rigid registration is known to decrease with increasing deformation between image datasets [47, 49, 50] 2) in contrast to other distortion correction methods that use prior knowledge, registration-based solutions do not guarantee improvement of geometric accuracy of corrected image datasets in every clinical case, as their iterative algorithms can converge locally or may simply be underconstrained in case of poor visibility of targets (homogeneous radiodensity of targets and surrounding tissues) in the CT images [50, 51]. Registration-based distortion correction should therefore only have a role as a supplementary tool after optimal prior knowledge-based distortion correction.

#### Distortion correction using image registration—Optional

- The use of registration-based distortion correction in addition to the minimum requirements may have a supplementary role in certain settings. *(Consensus 92%, abstention: 0%; Optional: 100%)*

## 2. Optimal sequence selection and optimization of sequence protocol parameters

**Fig. 2:**
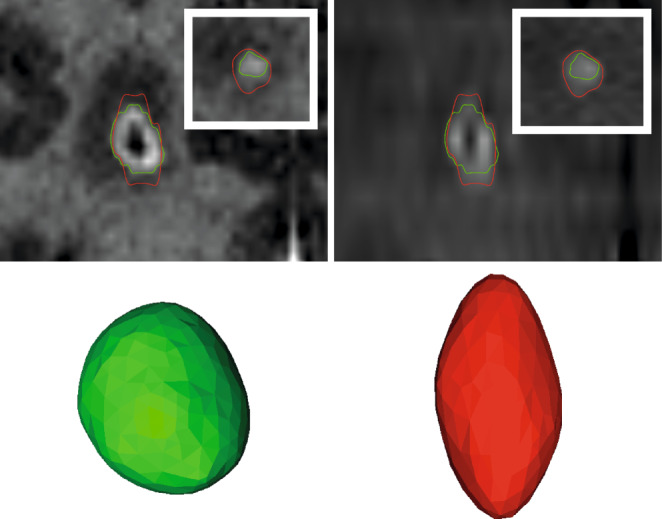
Importance of high-resolution 3D sequences for SRT treatment planning. *Left*: Coronal reconstruction of a high-resolution 3D T1w-IR-GE sequence of a small brain metastasis, tumor segmentation in *green* (GTV). *Right*: Coronal reconstruction of a low-resolution 2D T1-SE sequence, tumor segmentation in *red* (GTV). *Inset*: transversal view. *Bottom*: 3D rendering of the GTVs obtained from the high- and low-resolution T1 sequence. Note: Considerable difference in tumor size due to partial volume effects

Patients referred to stereotactic radiotherapy generally have their diagnosis established through diagnostic imaging beforehand. The primary objective of MRI for radiotherapy planning therefore is to accurately depict the tumor location and shape in three-dimensional space so that the gross tumor volume (GTV) can be precisely delineated. Generally, high-resolution isotropic 3D sequences are optimal for this task since they permit accurate multiplanar reconstruction and minimize over- or underestimation of the GTV because of partial-volume effects ([52, 53]; Fig. 2). Additionally, 3D sequences provide continuous imaging of the brain and targets without gaps and demonstrate lower susceptibility to distortions arising from *B*_0_ inhomogeneities compared to 2D sequences [12, 13, 17] (see above for a detailed discussion on optimizing sequence parameters for geometric accuracy). As a rule of thumb, the volumetric error will exceed 10%, if the target is visualized on less than 5 slices, which is particularly relevant for small targets such as brain metastases [53]. Partial volume effects because of large slice thickness will mostly result in an overestimation of the GTV volume. Moreover, thick slices and image gaps can also lead to an underestimation of tumor growth perpendicular to the imaging plane or miss small tumor parts [16, 53].

**Fig. 3:**
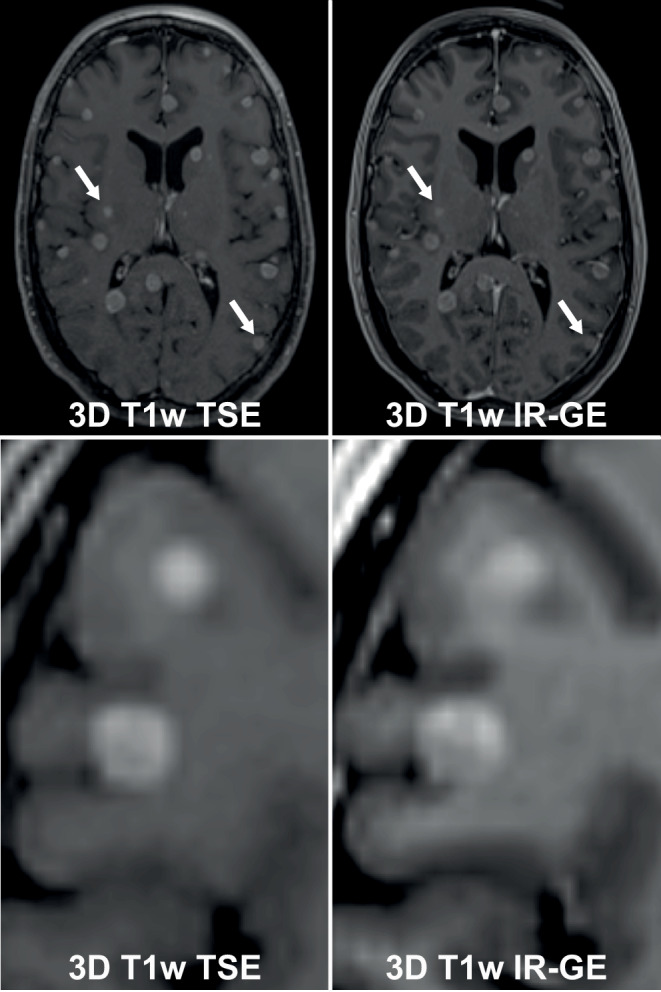
Difference of 3D T1w TSE and 3D T1w IR-GE sequences for treatment planning in brain metastases. Note: Improved target conspicuity in the 3D T1w TSE sequence, more prominent vessels and contrast between *grey* and *white* matter in the 3D T1 IR-GE sequence

The most used 3D-T1w MR sequences for brain tumors have been T1w inversion-recovery gradient echo (IR-GE) sequences such as the T1-MPRAGE [54, 55]. For cranial radiotherapy target volume delineation, however, multiple studies have recently suggested that 3D-T1w fast or turbo spin echo (FSE/TSE) sequences may in fact be superior to 3D-T1w IR-GE sequences (Fig. 3; [55–59]). Since 3D-T1w FSE/TSE sequences show less contrast between gray and white matter [55], they frequently improve the contrast ratio of contrast-enhancing lesions and low-contrasting brain parenchyma as background for targets. An equally beneficial effect for radiotherapy planning purposes is the suppression of vessels in 3D-T1w FSE/TSE sequences [58], which can facilitate discrimination of small tumors from transverse vessel cross-sections. For T1w IR-GE sequences, on the other hand, an additional well-described caveat is that they suffer from reduced visibility of enhancement when low contrast agent uptake is present, which could lead to an underestimation of the lesion boundaries [55, 60].

A further advantage of 3D-T1w FSE/TSE sequences over gradient echo sequences is that metal artefacts are significantly reduced, which is valuable when imaging patients with brain tumors who have shunts or surgical clips in place [23, 61]. However, 3D-T1w FSE/TSE sequences are more susceptible to motion compared to gradient echo sequences and may introduce artifacts due to the reliance on high turbo factors [62].

Despite, diagnostic imaging generally has been performed before MRI simulation, the SRT planning MRI may also need to address diagnostic uncertainty, as targets and patient anatomy may undergo important changes and new complications, like intratumoral bleeding, may arise [8, 63]. In some cases, diagnostic uncertainty may even be the leading factor affecting treatment precision. Neuroradiologic and radiologic sequence protocol recommendations addressing individual patient factors are therefore important to incorporate in addition to the following general guidance.

### Optimal sequence selection and optimization of sequence protocol parameters—Minimum requirements

- MRI protocols must be used that include at least one 3D sequence (e.g., 3D-T1w) with a sufficient signal-to-noise ratio (SNR) for target delineation. *(Consensus: 100%, abstention: 0%; Minimum requirement: 100%)*
- Standardized MRI protocols must be set up and used for cranial stereotactic treatment planning indications. These standardized protocols must be characterized by a unique and easily understandable study description (e.g., “RT treatment planning—brain metastases”). *(Consensus: 100%, abstention: 0%; Minimum requirement: 100%)*
- The main 3D sequence must be isovolumetric with a voxel size of ≤ 1 mm^3^. *(Consensus: 100%, abstention: 0%; Minimum requirement: 100%)*
- Choose the 3D-T1w sequence that provides the best target conspicuity and the most accurate characterization of 3D tumor boundaries. For a substantial fraction of patients, treatment indications and MR scanners, 3D-T1w FSE/TSE sequence protocols are to be preferred over 3D-T1w IR-GE sequence protocols [55, 59, 64]. If multiple 3D sequence protocols for target delineation are acquired, generally, the gross target volume should encompass the extent of the tumor in all 3D sequences. *(Consensus: 100%, abstention: 0%; Minimum requirement: 85%, Additional recommendation: 15%)*

### 2.1. Indication-specific considerations for sequence selection

Many treatment indications for cranial SRT benefit from the acquisition of additional 3D sequences for high-resolution target delineation or are not properly visualized on contrast-enhanced 3D-T1w sequences alone. These for example include 3D-True FISP/Dual Excitation (Siemens: 3D-CISS/GE: 3D-FIESTA-C) or 3D-T2w FSE/TSE sequences for cranial nerve targets and vestibular schwannomas [65–69]. Recommended sequence parameters and protocols for SRT treatment planning are shown in Tables 1 and 2. As some treatment planning systems still exclusively accept strictly transversal image datasets, reformatting or even acquisition of all sequences in transversal orientation may be an additional requirement for some centers. As described above, individual neuroradiologic and radiologic sequence protocol recommendations should be incorporated and especially complex clinical cases require diagnostic input for optimal target volume definition.

**Table 1:** MRI Sequence parameters recommended for cranial stereotactic treatment planning at 1.5 T

	3D-T1w FSE/TSE	3D-T1w IR-GE	3D-T2-FLAIR FSE/TSE	3D-T2w FSE/TSE	3D-True FISP/Dual Excitation
*Type*	3D	3D	3D	3D	3D
*Orientation*	Transversal or Sagittal	Transversal or Sagittal	Transversal or Sagittal	Transversal	Transversal
*TE*	Minimum^a^	Minimum^a^	e.g., 374 ms	e.g., 182 ms (heavily T2w) e.g., 92 ms (moderately T2w)	e.g., 2.45 ms
*TR*	e.g., 550–750 ms	e.g., 2100–2200 ms	e.g., 7000 ms	e.g., 1200 ms	e.g., 5.47 ms
*TI*	–	e.g., 900–1100 ms	e.g., 2050 ms	–	–
*Acq. matrix*	≥ 256 × 256	≥ 256 × 256	≥ 256 × 256	≥ 256 × 256	≥ 256 × 256
*Acq. in-plane resolution*	≤ 1 × 1 mm	≤ 1 × 1 mm	≤ 1 × 1 mm	≤ 0.7 × 0.7 mm	≤ 0.7 × 0.7 mm
*Slice thickness*	≤ 1 mm	≤ 1 mm	≤ 1 mm	≤ 0.7 mm	≤ 0.7 mm
*Fat saturation*	Optional	–	–	–	–
*Post-contrast interval*	≥ 5 min	≥ 5 min	–	–	–
*GNL Distortion correction*	Vendor 3D ± in-house^b^	Vendor 3D ± in-house^b^	Vendor 3D ± in-house^b^	Vendor 3D ± in-house^b^	Vendor 3D ± in-house^b^
*Shimming of B*_*0*_* inhomogeneities*	Patient-specific active shimming	Patient-specific active shimming	Patient-specific active shimming	Patient-specific active shimming	Patient-specific active shimming
*Readout bandwidth*	≥ 440 Hz (≥ 660 Hz recommended)	≥ 440 Hz (≥ 660 Hz recommended)	≥ 440 Hz (≥ 660 Hz recommended)	≥ 440 Hz (≥ 660 Hz recommended)	≥ 440 Hz (≥ 660 Hz recommended)
*Interval to treatment*	≤ 14 days (≤ 5 days recommended)	≤ 14 days (≤ 5 days recommended)	≤ 14 days (≤ 5 days recommended)	≤ 14 days (≤ 5 days recommended)	≤ 14 days (≤ 5 days recommended)

*TE* echo time, *TR* repetition time, *TI* inversion time, *Acq.* Acquisition, *GNL* gradient non-linearity, *B*_*0*_ main magnetic field

^a^Use the minimum possible TE interval, in general the TE interval should be ≤ 10 ms

^b^Inhouse GNL distortion correction refers to obtaining a deformation field via phantom measurements to correct residual distortion after vendor 3D GNL distortion correction

^c^If patient-specific active shimming is not available, *B*_0_ inhomogeneities during patient image acquisition should be characterized and corrected with advanced methods if relevant (see text)

**Table 2:** Recommended planning MRI sequence protocols for important stereotactic radiotherapy treatment indications

Brain metastases	Meningioma	Vestibular schwannoma	Pituitary adenoma	Trigeminal neuralgia	AVM	Glomus tumors
2D-T1w pre (*optional: 3D-T1w pre*)	3D-T1w pre	2D-T1w pre (*optional: 3D-T1w pre*)	2D-T2w FSE/TSE cor	3D-T1w pre	2D-T1w pre (*optional: 3D-T1w *GE *pre*)	3D-T1w pre
**Contrast administration**	**Contrast administration**	**Contrast administration**	2D-T2-FLAIR tra	3D-TOF	3D-TOF	**Contrast administration**
2D-T2-FLAIR tra	2D-T2-FLAIR tra	2D-T2-FLAIR	3D-T1w pre	**Contrast administration**	3D-T2w FSE/TSE	3D-T2w FSE/TSE / 3D-True FISP-Dual Excitation
3D-T1w post early	3D-T1w post	3D-T1w post	**Contrast administration**	3D-T2w FSE/TSE / 3D-True FISP-Dual Excitation	**Contrast administration**	3D-T1w post
(*Optional: 3D-T1w post late*)	Subtraction 3D-T1w post − T1w pre	3D-T2w FSE/TSE / 3D-True FISP-Dual Excitation	2D-T1w dynamic cor	3D-T1w post	3D-T1w GE post	Subtraction 3D-T1w post − T1w pre
			3D-T1w post		*(Optional: Subtraction* *3D-T1w GE post* *−* *pre)*	
			Subtraction 3D-T1w post − T1w pre

**Table 3:** Test frequencies and intervention thresholds for regular quality assurance

Parameter	Minimum requirement	Additional recommendation	Optional
End-to-end test including MRI simulation	Yearly, after commissioning and after changes to the SRT treatment planning chain in accordance with DIN 6864‑1	–	–
Residual gradient non-linearity-related distortions after vendor 3D correction	Characterize using phantom measurements at the time of commissioning, after scanner upgrades, repairs or maintenance, and at least in yearly intervals. Maximum amount of distortion obtained via phantom measurements for the field-of-view of a typical head MRI scan (sphere of 25 cm diameter) must not exceed 1 mm	In addition to minimum requirements: Characterization of residual gradient non-linearity related distortions in at least monthly intervals using phantom measurements	In addition to additional recommendations: Correction of residual distortions ≤ 1 mm based on phantom measurements
Main magnetic field (B0) homogeneity	Characterize after installation (baseline), after scanner upgrades, repairs or maintenance, and at least in yearly intervals, as detailed in sources such as [32] and [33]. If necessary, arrange for repairs to maintain field homogeneity and ensure necessary geometric accuracy	–	In addition to minimum requirements: Individually characterize main magnetic field inhomogeneities from system imperfections and susceptibility-related inhomogeneities for patients undergoing MRI simulation by using B0 mapping. Perform individual corrections based on individual B0 mapping or reverse gradient methods
Screening for metallic objects	Daily check the scanner bore for small metallic objects; Check gradient echo-based localizer images for metal artifacts in every patient	–	–
Registration algorithm	Registration error within ≤ 1 mm for registration of phantoms at commissioning	–	–
Image quality for flexible coil systems	–	–	Monthly

#### Brain metastases

A high-resolution contrast-enhanced 3D-T1w sequence should be the main sequence for target delineation in brain metastases. Care must be taken to ensure that sufficient time is allowed between contrast administration and sequence acquisition for contrast uptake to occur, enabling all lesions to be clearly visible and the tumor boundaries to be accurately identified (see below). Therefore, a T2-FLAIR sequence should be acquired as a “spacer” between contrast administration and acquisition of the main 3D-T1w sequence. The T2-FLAIR sequence does not add much information on the configuration of the target volume for individual lesions, however it provides complementary diagnostic information, e.g., on the presence of perifocal edema, which indicates an elevated risk for lesion shift [70] and informs the need for corticosteroid dose adaption [43]. A second late contrast-enhanced 3D-T1w sequence can be acquired 15–20 min after the first contrast administration with or without application of a second dose of contrast media to further improve the visibility of metastases and lesion conspicuity [71–73]. Prior to contrast administration a T1w sequence should be acquired to discriminate contrast enhancement from other causes for T1w hyperintensity like bleeding or melanin-containing metastases [43].

#### Meningiomas

Meningiomas are typically strongly contrast-enhancing and are therefore best delineated using 3D-T1w sequences [74]. Subtraction sequences between pre- and postcontrast 3D-T1w sequences can help with tumor delineation near blood vessels and sinuses [75]. Due to their frequently peripheral location at the skull convexity, geometric accuracy is of particular importance in meningiomas.

#### Vestibular schwannomas

Vestibular schwannomas exhibit intense contrast-enhancement and are well demarcated on 3D-T1w [76]. A 3D-True FISP/Dual Excitation (Siemens: 3D-CISS/GE: 3D-FIESTA-C) or 3D-T2w FSE/TSE sequence provides additional information for target volume definition. In these heavily T2-weighted sequences vestibular schwannomas and accompanying cranial nerves appear hypointense in front of the bright CSF background [77]. 3D-True FISP/Dual Excitation (Siemens: 3D-CISS/GE: 3D-FIESTA-C) and 3D-T2w FSE/TSE sequences enable a particularly high in-plane and slice resolution, which additionally improves the accuracy of target volume delineation for these generally small tumors. Moreover, these sequences are very well suited for delineation of the cochlea, the semi-circular canals and cranial nerve OARs [77].

#### Pituitary adenomas

Pituitary adenomas typically appear hypointense in relation to residual normal pituitary gland tissue. 3D-T1w sequences alone are frequently not adequate for optimal target volume definition of pituitary adenomas. For accurate delineation of pituitary adenomas, it’s typically beneficial to additionally use 3D-T1w subtraction sequences and dynamic T1w sequences, which offer sequential image datasets at various stages of contrast enhancement [78].

#### Trigeminal neuralgia

High-resolution strongly T2-weighted 3D-True FISP/Dual Excitation (Siemens: 3D-CISS/GE: 3D-FIESTA-C) or 3D-T2w FSE/TSE should be the main sequences for target volume delineation in trigeminal neuralgia. These sequences depict the hypointense trigeminal nerve fibers against the background of the bright CSF [77]. Due to their high resolution, they are also well suited for target delineation in case of nerval atrophy. 3D-T1w sequences can additionally help with target volume delineation for trigeminal neuralgia and 3D-TOF sequences can depict associated vessels for sparing [79].

#### Glomus tumors

Glomus tumors are well vascularized benign neoplasms that typically show intense homogeneous contrast-enhancement and are well visualized on 3D-T1w FSE/TSE sequences [80, 81]. The main challenges in MRI for radiotherapy target volume definition is the discrimination from accompanying hyperintense vessels and the occasionally substantial caudal extension through the jugular foramen along the jugular vein [81, 82]. Substantial inferior extension along the carotid sheath requires MR imaging with a sufficiently large craniocaudal field-of-view. As the neck anatomy can be affected by substantial non-rigid tissue deformation with different positioning and 3D-T1w FSE/TSE sequences are susceptible to motion in the neck, imaging in treatment position with mask immobilization can be particularly beneficial in glomus tumors. Distinguishing glomus tumor from surrounding vascular structures can be improved using subtraction sequences [75].

#### Arteriovenous malformation

Arteriovenous malformations are usually best visualized on 3D-TOF angiography [83]. In addition, 3D-T2w FSE/TSE and contrast-enhanced 3D-T1w are complementary useful for optimal target delineation. With vascular targets, 3D-T1w GE should be used instead of 3D-T1w FSE/TSE sequences because of the improved depiction of vessels in 3D-T1w GE sequences [58]. On 3D-T2w sequences, arteriovenous malformations appear hypointense due to the associated flow void and can thus be discriminated from the surrounding brain and CSF [84].

#### Indication-specific considerations for sequence selection—Minimum requirements:

**Fig. 4:**
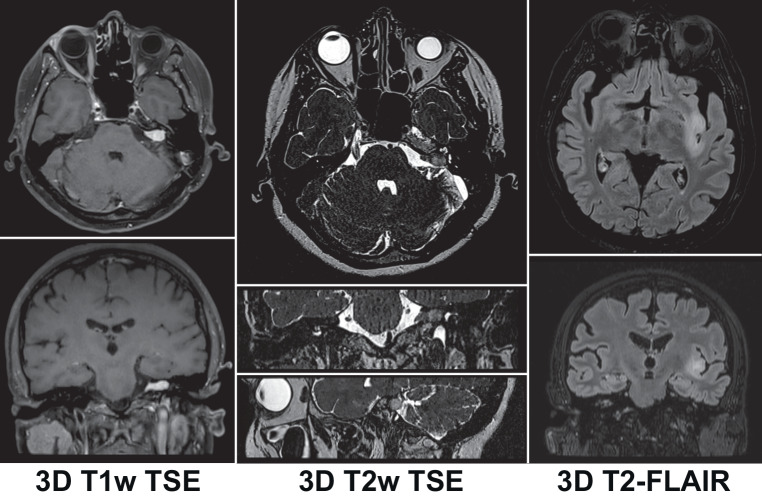
3D MRI sequences for intracranial SRT treatment planning. *Left*: 3D T1w TSE sequence in a patient with vestibular schwannoma. *Center*: 3D T2w TSE sequence in the same patient, *Right*: 3D T2-FLAIR sequence. Note: high-resolution multiplanar reconstruction

MRI protocols must be used that include 3D sequence protocols for all required image contrasts (e.g., 3D-T1w, 3D-T2w, 3D-T2-FLAIR) needed for target and organs-at-risk *delineation *(Fig. 4). *(Consensus: 92%, abstention: 8%; Minimum requirement: 100%).*

## 3. Contrast enhancement

Additional critical parameters that can affect the delineation of lesions in T1-based MR sequences include the dose of gadolinium-based contrast agent (GBCA), as well as the time interval between contrast application and measurement [52, 71–73]. In a study by Yuh et al., early imaging (10 min) and late imaging (20 min) following standard dose gadoteridol were compared [71]. After 10 min, 40.6% of metastases < 5 mm were visible, while 75.0% were visible after 20 min. Only after an additional bolus of double-dose gadoteridol, the remaining lesions were evident, demonstrating the importance of increasing the doses of GBCA [71]. Likewise, Baleriaux et al. found an increase in the number of metastases and a better visual conspicuity of the lesions with increasing cumulative doses of gadobenate dimeglumine over several sequential injections [72]. Similarly, Kushnisky et al. found more brain metastases at 15 min after GBCA administration compared to 5 min. They also notably found an increase in metastasis volume after 10 min compared to 5 min, as well as 15 min compared to 10 min postcontrast [73].

### Contrast enhancement—Minimum requirements

- For intraaxial tumors, the time interval between contrast administration and the start of the acquisition of the main T1w sequence should be at least 5 min (see discussion in main text). *(Consensus: 100%, abstention: 0%; Minimum requirement: 100%)*

### Contrast enhancement—Optional

- Given the improved lesion conspicuity with increased contrast dose, administration of double-dose contrast may be considered in specific circumstances, if the individual benefit of improved tumor delineation for treatment planning clearly outweighs individual GBCA-associated risks. *(Consensus: 100%, abstention: 15%; Additional recommendation: 9%, Optional: 91%)*
- An additional delayed T1w sequence protocol may be acquired 15–20 min after the first contrast administration, with or without repeated contrast administration to improve target conspicuity and depiction of target boundaries. *(Consensus: 92%, abstention: 8%; Optional: 100%)*

## 4. Time interval between MRI simulation and treatment delivery

**Fig. 5:**
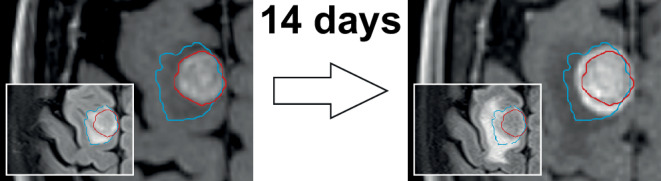
Importance of minimizing the time interval between imaging and SRT. Considerable tumor growth in a patient with brain metastasis in only 14 days (3D T1w). *Inset*: Increase in perifocal edema (T2-FLAIR) causing a shift in tumor position

The interval between MRI and treatment delivery is one of the most crucial parameters for treatment precision ([7, 8]; Fig. 5). The importance of simulation MRI datasets being up-to-date is especially high for rapidly growing tumors such as brain metastases [8, 63] that are also often associated with fluctuating amounts of surrounding edema [85, 86].

This was confirmed by Seymour et al., who found a large detriment in local control following radiosurgery in brain metastases, if the interval between MRI and radiosurgery was greater than or equal to 14 days (local control 56% vs. 95% at 6 months post-SRS) [7]. Subsequently, Salkeld et al. even reported relevant differences with imaging intervals ≤ 7 days before SRS. In this study, 41% of patients with an interval ≤ 7 days required a change in radiooncologic management, whereas even 78% of patients required changes if the delay exceeded 7 days. Replanning was performed most frequently due to an increase in the tumor or resection cavity volume [8, 87]. In a retrospective review of 101 patients and 531 brain metastases, Kutuk et al. confirmed that changes in tumor size and spatial position occurred as a function of time, with the risk of requiring a margin beyond 1 mm increasing every day by 5% [88]. Therefore, the interval between imaging and treatment delivery should be as short as possible.

### Repeated MR imaging during fractionated stereotactic radiotherapy

During fractionated stereotactic radiation therapy, brain metastases and malignant primary brain tumors can undergo profound changes due to transient swelling, changes in perifocal edema, and incipient treatment effect. An analysis by Hessen et al. evaluated the significance of repeated MRI scans during fractionated SRT for 18 brain metastases and 20 resection cavities. In cases with in situ brain metastases, there was a reduction in PTV coverage of up to 34.8%, while postoperative cases were less affected (up to −4.5% in PTV coverage) [89]. Importantly, as only 3–5 fractions were employed in the study by Hessen et al., even more pronounced changes would be expected with more prolonged fractionation schemes. Uto et al. investigated interfractional target changes in 27 brain metastases (23 brain metastases) during 13-fraction FSRT based on a mid-treatment MRI acquired after a median of 6 days. Compared to the baseline MRI, the mid-treatment GTV had increased by more than 20% in 5 lesions and decreased by more than 20% in another 5 tumors. Interestingly, in 15 out of 27 brain metastases the initial PTV did not encompass the entire mid-treatment GTV [90]. More recently, similar findings were reported in a series of 114 brain metastases (66 patients) treated with 10 to 20 fractions of gamma knife FSRT. After a median of 7 days between the initial simulation MRI and the interfractional MR imaging, interfractional changes between −48 to 72% in tumor volume were observed and 29% of lesions showed significant volume changes (defined as ≥ +20% or ≤ −20% change in volume). Beyond volumetric changes, 85% of treatment plans needed to be modified, because of the information provided by the mid-treatment MRI [91].

### Time interval between MRI simulation and treatment delivery—Minimum requirements

- The time interval between the MRI simulation and the administration of treatment must not be larger than 14 days. *(Consensus: 100%, abstention: 8%; Minimum requirement: 100%)*

### Time interval between MRI simulation and treatment delivery—Additional recommendations

- In brain metastases and primary brain tumors CNS-WHO-grade 2–4, the time interval between MR simulation and treatment delivery should not be greater than 5 days. *(Consensus: 100%, abstention: 8%; Minimum requirement: 17%, additional recommendation: 83%)*

### Time interval between MRI simulation and treatment delivery—Optional

- Consider, performing an additional simulation MRI for adaption of target volumes every 5 fractions in fractionated stereotactic radiotherapy (≤ 12 fractions). *(Consensus: 100%, abstention: 0%, Optional: 100%)*

## 6. Image registration and imaging in SRT position (Fig. 6)

The simulation MRI for SRT is most frequently acquired in a diagnostic head coil and subsequently rigidly coregistered to the planning CT obtained in the treatment position with mask immobilization. Therefore, it is an essential prerequisite that the registration of the simulation MRI to the geometry of the planning CT in treatment position is as accurate as possible. In general, rigid registration that involves translations and rotations only is the optimal type of registration for cranial SRT. In case of multiple target volumes, multiple individual registrations in a reduced volume of interest covering each lesion separately should be considered [50]. The software used for image registration must be properly commissioned and validated followed by a quality assurance program for image registration e.g., according to AAPM Task Group report 132 [50]. This involves quantitative validation of registration accuracy with phantoms [50]. For cranial SRT, the employed rigid registration algorithm should have a registration error within 1 mm in registration phantoms [50, 92, 93]. Moreover, in daily clinical practice every MRI-CT registration for treatment planning must be verified qualitatively by a clinical expert with board certification. Software manufacturers developing treatment planning systems for cranial stereotactic radiotherapy are encouraged to offer a quantitative assessment of the registration method’s accuracy using an independent approach from the registration algorithm (e.g., employing an automatic landmark placement technique when the registration is carried out by optimizing the normalized mutual information). For the brain, in individual publications the accuracy of normalized-mutual-information based MRI-CT coregistration has been reported to be ≤ 0.5 mm in plane and ≤ 1 mm along the Z‑axis for CT slices with a thickness between 2 and 3 mm and a 2 mm 3D-T1w IR-GE sequence. However, the registration uncertainty increased by a factor of 2–3 when a 2D-SE sequence with a slice thickness of 5 mm was registered to a 5-mm planning CT [92]. More generally, registration accuracies of ~ 2 mm are reported [94, 95]. This finding highlights the significance of using high-resolution pairs of MRI and CT datasets to ensure adequate registration accuracy. MRI datasets used for registration should be 3D sequences, with equidistant slice planes and without gaps. MRI datasets must also be free from distortions, motion, folding, and metal artifacts and errors in planning CT datasets have to be minimized for optimal registration accuracy [50]. Due to the long scanning times with MRI, it is also important to exclude motion between individual sequences that are not addressed by registration. Many treatment planning systems automatically propagate the transformation from the registration of one MRI sequence to all other sequences in the study, which may lead to poor registration accuracy in case of intermediate motion. It is therefore important to individually verify the registration accuracy of every MRI series used for treatment planning.

**Fig. 6:**
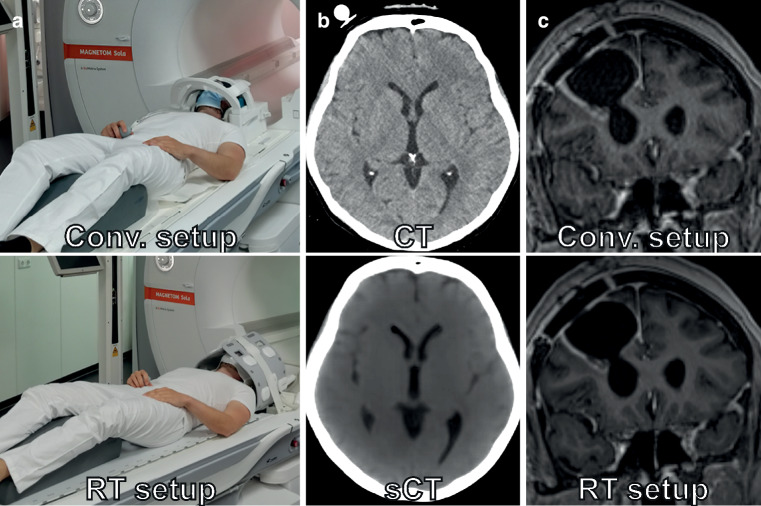
SRT treatment setup with mask immobilization. **a** Conventional imaging setup in diagnostic head coil (*top*) vs. imaging setup in RT treatment position with mask immobilization enabled by flexible receiver coils (*bottom*). **b** Conventional planning CT (*top*) vs. Synthetic CT (sCT) reconstructed from MRI sequences (*bottom*). **c** Less motion artifacts with mask immobilization in the RT imaging setup (*bottom*) compared to the standard imaging setup without mask immobilization (*top*)

Acquiring planning MRI datasets in the treatment position may improve the accuracy of rigid registration, as planning MRI and CT images are acquired in a similar anatomical configuration [30, 93, 96]. Multiple groups have reported imaging setups to acquire simulation MRIs in the treatment position with mask immobilization [24, 96, 97]. Flexible coil setups have been developed most frequently for this purpose, as most stereotactic mask systems will not fit into routine radiological head coils [30, 97–99]. Masitho et al. evaluated the registration accuracy of three treatment planning systems for coregistering planning MRIs acquired in a diagnostic head coil and in radiotherapy treatment position with mask immobilization to a common planning CT. Generally, the software used for registration had the most significant impact on the accuracy of the registration. However, for less optimal registration methods, registration accuracy was significantly improved if simulation MRIs had been performed in the treatment position [93]. For targets near the foramen magnum, performing the planning MRI in treatment position could be more beneficial due to slight deformation occurring with different extension of the occipito-atlanto-axial joint complex [61]. In addition to slightly improving the accuracy of the registration, imaging in the treatment position with mask immobilization significantly decreases motion artifacts, which may help with the precise definition of the target volume [97, 99]. However, usually some decrease in image quality is expected from imaging with a flexible coil setup in treatment position as compared to using dedicated diagnostic head coils, which may need to be compensated with a longer measurement time [99]. Since flexible coils are more prone to mechanical damage, monthly quality assurance is recommended as proposed in the AAPM TG 284 report [10]. The procedure can be combined with validating geometric accuracy, gain, and spatial resolution which are part of quality assurance recommendations of the ACR [32].

Acquiring planning MRI studies in the treatment position with mask immobilization becomes a necessity when using an MR-only workflow [100]. Synthetic CT, involving the computation of synthetic CT images from one or multiple MR sequences, eliminates the requirement for an additional planning CT, thereby theoretically removing any registration uncertainties [101]. Current methods provide reasonable dosimetric results in most standard situations and dosimetric differences < 1% have been reported for deep learning methods recently [100, 102]. It has also been reported that sCTs provide adequate means for positioning the patient at the linear accelerator, i.e., as reference for registering the CBCT [103, 104] as well as 2D/2D based positioning systems [93]. However, when synthetic CT is employed in an MR-only workflow, care must be taken that the treatment position is exactly reproduced on the MR scanner as the synthetic CT will serve both as a foundation for dose calculation as well as a reference for image guidance at the radiotherapy treatment unit [100]. It is equally important to ensure that no changes in patient position occur between acquisition of the dedicated synthetic CT sequences and the MR sequences used for target delineation that are not addressed by registration. It is therefore recommended that prior to clinical implementation of an MR-only workflow, experience with MR simulation has been established, with optimized sequence selection and protocol parameters as well as by optimal quality assurance using the advanced recommendations as described above.

### Image registration and imaging in SRT position—Minimum requirements

- When registering simulation MRI datasets to a planning CT, both the planning CT and the MRI dataset, i.e., both registration pairs, must have a slice thickness of ≤ 1 mm. *(Consensus: 100%, abstention: 0%; Minimum requirement: 92%, additional recommendation: 8%)*
- A proper registration algorithm, commissioned, and validated for stereotactic radiotherapy/radiosurgery followed by expert correction and verification must be used. *(Consensus: 100%, abstention: 0%; Minimum requirement: 100%)*
- With quantitative validation at commissioning, the registration algorithm must achieve a registration error within ≤ 1 mm for registration of phantoms. *(Consensus: 100%, abstention: 0%; Minimum requirement: 100%)*
- Artifacts that could impair registration must be minimized: Set a sufficient phase oversampling factor to avoid folding artifacts, minimize distortions in MRI, minimize metal artifacts in MRI (e.g., by using 3D-T1w FSE/TSE instead of 3D-T1w GE sequence protocols) and minimize artifacts in planning CTs (e.g., minimize metal artifacts from dental fillings). *(Consensus: 100%, abstention: 0%, Minimum requirement: 92%, additional recommendation: 8%)*
- Motion artifacts inside the diagnostic head coil must be minimized by proper use of cushions and foam elements. *(Consensus: 100%, abstention: 0%; Minimum requirement: 92%, additional recommendation: 8%)*
- In daily clinical practice, registration quality for each treatment planning registration must be verified qualitatively by a board-certified physician or medical physics expert with experience in cranial stereotactic radiotherapy. This verification should be performed using an overlay method (e.g., alpha blending with or without varying color schemes for both datasets, a checkerboard pattern, and/or “spy glass” tools), with dynamic assessment of registration accuracy (e.g., by blending between datasets or by moving the checkerboard pattern and “spy glass” tool). Site-specific recommendations can be found in [21], for example. *(Consensus: 100%, abstention: 0%; Minimum requirement: 100%)*
- When using multiple sequences for treatment planning, registration accuracy must be individually verified for each sequence used for treatment planning, because of the risk of motion between sequences. *(Consensus: 100%, abstention: 0%; Minimum requirement: 100%)*

### Image registration and imaging in SRT position—Optional

- Consider, acquiring simulation MRIs in the treatment position with mask immobilization to improve registration accuracy and to reduce/eliminate motion artifacts. Sufficient image quality must be ensured when acquiring simulation MRIs in the treatment position with a flexible coil setup. *(Consensus: 85%, abstention: 0%; Additional recommendation: 9%, Optional: 91%)*
- Especially consider acquisition in the treatment position with mask immobilization if nonrigid tissue deformations are expected between the treatment position and the diagnostic imaging position (e.g., for targets near the foramen magnum and if rigidity of the skull is significantly impaired after surgery). *(Consensus: 92%, abstention: 0%; Additional recommendation: 8%, Optional: 92%)*
- Consider, performing monthly checks on image quality for flexible coil systems employed for stereotactic radiotherapy simulation [10]. *(Consensus: 85%, abstention: 0%; Additional recommendation: 9%, optional: 91%)*
- Use of synthetic CT and an MR-only workflow may be considered to exclude MRI-CT registration uncertainties. If an MR-only workflow is used, it must be ensured that synthetic CT datasets can be used for both treatment planning and image guidance. In addition, motion between MRI sequences used for synthetic CT calculation and sequences used for target delineation must be excluded or addressed by registration. *(Consensus: 92%, abstention: 0%; Minimum requirement: 8%, additional recommendation: 8%, optional: 83%)*

## Conclusion

Accurate MRI simulation is a critical basis for precise treatment in cranial stereotactic radiotherapy and an integral part of stereotactic radiotherapy treatment planning. This report recommends measures and procedures to optimize MRI sequence protocols, to verify spatial precision and to optimize the clinical workflow to secure the fidelity and spatial precision of MR images in SRT. Broadly implementable minimum requirements provide major improvements in accuracy, while additional recommendations and options allow advanced centers to further optimize MR-based treatment planning. This guideline and especially the topics of repeated imaging during fractionated treatment, as well as the role of MR-only treatment planning, will require an update when more evidence becomes available.
